# Comparison of revascularization and conservative treatment for hemorrhagic moyamoya disease in East Asian Countries: a single-center case series and a systematic review with meta-analysis

**DOI:** 10.3389/fneur.2023.1169440

**Published:** 2023-06-02

**Authors:** Xiang-Hua Zhang, Jun-Hua He, Xiang-Sheng Zhang, Jing Zhang, Cheng-jun Wang, Yi-Peng Dong, Wu Tao

**Affiliations:** ^1^Department of Neurosurgery, Beijing Friendship Hospital Affiliated With Capital Medical University, Beijing, China; ^2^Department of Neurosurgery, Zhejiang Provincial Tongde Hospital, Hangzhou, China

**Keywords:** revascularization, conservative, rebleeding, ischemic, mortality, moyamoya disease, hemorrhagic

## Abstract

**Objective:**

The optimal treatment approach for hemorrhagic moyamoya disease (HMMD) remains a topic of debate, particularly regarding the comparative efficacy of revascularization versus conservative treatment. Our study, which included a single-center case series and a systematic review with meta-analysis, aimed to determine whether surgical revascularization is associated with a significant reduction in postoperative rebleeding, ischemic events, and mortality compared to conservative treatment among East Asian HMMD patients.

**Methods:**

We conducted a systematic literature review by searching PubMed, Google Scholar, Wanfang Med Online (WMO), and the China National Knowledge Infrastructure (CNKI). The outcomes of surgical revascularization and conservative treatment, including rebleeding, ischemic events and mortality, were compared. The authors' institutional series of 24 patients were also included and reviewed in the analysis.

**Results:**

A total of 19 East Asian studies involving 1,571 patients as well as our institution's retrospective study of 24 patients were included in the study. In the adult patients-only studies, those who underwent revascularization had significantly lower rates of rebleeding, ischemic events, and mortality compared to those who received conservative treatment (13.1% (46/352) vs. 32.4% (82/253), *P* < 0.00001; 4.0% (5/124) vs. 14.9% (18/121), *P* = 0.007; and 3.3% (5/153) vs. 12.6% (12/95), *P* = 0.01, respectively). In the adult/pediatric patients' studies, similar statistical results of rebleeding, ischemic events, and mortality have been obtained (70/588 (11.9%) vs. 103/402 (25.6%), *P* = 0.003 or <0.0001 in a random or fixed-effects model, respectively; 14/296 (4.7%) vs. 26/183 (14.2%), *P* = 0.001; and 4.6% (15/328) vs. 18.7% (23/123), *P* = 0.0001, respectively).

**Conclusion:**

The current single-center case series and systematic review with meta-analysis of studies demonstrated that surgical revascularization, including direct, indirect, and a combination of both, significantly reduces rebleeding, ischemic events, and mortality in HMMD patients in the East Asia region. More well-designed studies are warranted to further confirm these findings.

## 1. Introduction

Moyamoya disease (MMD) is a chronic idiopathic condition that was first described by Taceuchi and Shimizu in 1957 (1). This condition is characterized by nonatherosclerotic progressive stenosis or occlusion of the bilateral supraclinoidal internal carotid arteries and the development of an abnormal collateral vascular network at the base of the brain. This disorder is especially prevalent in East Asian populations, mainly Japan, Korea, and China, and the reported prevalence of MMD is 10.5/100,000 individuals in Japan (2), 16.1/100,000 in South Korea (3), and 3.92/100,000 in China (4), respectively. In MMD, intracranial hemorrhage occurs more frequently in adult patients than in children (5), especially in adults older than 40 years. Surgical revascularization, including direct bypass, indirect bypass, and combinations of both, has proven to be effective in improving outcomes for patients with ischemic MMD (6, 7). However, whether surgical revascularization could reduce the long-term risks of recurrent hemorrhage (8), ischemic events, and mortality in HMMD patients remains controversial. The purpose of this study was to determine whether surgical revascularization reduces the risk of recurrent hemorrhage, ischemic events, and mortality in East Asian HMMD patients.

## 2. Materials and methods

### 2.1. Literature search

This study was conducted according to the Preferred Reporting Items for Systematic Reviews and Meta-Analyses (PRISMA) guidelines (9). A comprehensive literature search was performed on PubMed, Google Scholar, Wanfang Med Online (WMO), and the China National Knowledge Infrastructure (CNKI) for studies on HMMD published before 1 January 2023. The terms “moyamoya disease,” “hemorrhagic,” “conservative,” and “revascularization” were used as keywords in searching the abovementioned databases. Other relevant publications were identified by examining the references included in the study.

### 2.2. Inclusion and exclusion criteria

The inclusion criteria were as follows: (1) HMMD patients; (2) adult or pediatric patients; (3) the study including both surgical and conservative treatment groups; (4) articles written in English or Chinese.

The exclusion criteria were as follows: (1) system review articles, case reports, and editorials; (2) moyamoya syndrome; (3) other surgical treatment modalities (such as aneurysm clip or coil procedure, hematoma evacuation, and so on); and (4) without detailed outcomes for revascularization procedures and conservative treatment.

### 2.3. Data extraction

**Figure 1:**
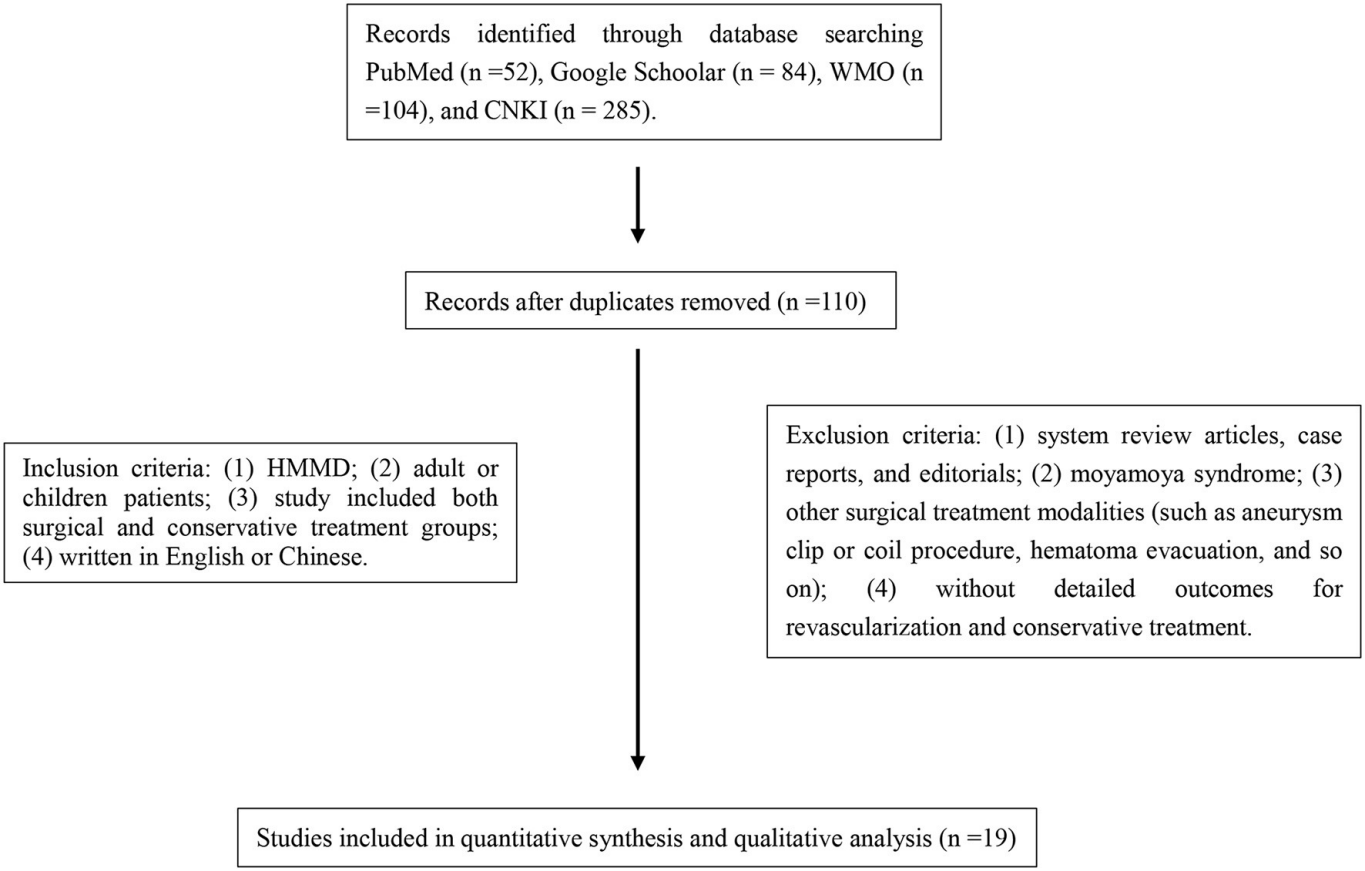
Flow diagram of the literature search strategy.

A total of 525 studies were identified through a search of PubMed (n =52), Google Scholar (n = 84), WMO (n =104), and CNKI (n = 285), among which 110 studies were first excluded due to duplicate citations. According to the inclusion and exclusion criteria, 19 studies of the remaining 415 were finally included in the systematic review and meta-analysis (Figure 1), (Tables 1, 2).

**Table 1:** Study characteristics and rebleeding in conservative and revascularization groups.

**References**	**Country**	**Study design**	**Sample size(n) child/adult**	**Age (mean ys.) male/female**	**NOPs (RV/RB)**	**NOPs (CO/RB)**
Fujii et al. (10)	Japan	RC	290	NA	152/29	138/39
			NA	NA		
Ikezaki et al. (11)	Japan	RC	197	NA	80/15	117/19
			NA	NA		
Yoshida et al. (12)	Japan	RC	28 (7 lost to follow-up)	39.26 ± 15.7	8/1	13/5
			2/26	4/24		
Kawaguchi et al. (13)	Japan	RC	22	43	11/2	11/2
			0/22	7/15		
Duan et al. (14)	China	RC	61	37.6	59/0	2/2
			4/57	29/32		
Zhao et al. (15)	China	RC	23	S 46.42, NS 46.6	10/1	13/1
			0/23	12/11		
Choi et al. (16)	Korea	RC	44	44.9 (17–65)	35/6	9/4
			0/44	18/26		
Liu et al. (17)	China	RC	97	S 33, NS30	54/4	43/17
			6/91	33/64		
Miyamoto et al. (18)	Japan	MPRCT	80	S42.5, NS 41.4	42/5	38/12
			0/80	24/56		
Chen et al. (19)	China	RC	82	Child 9.6/adult 37.2	48/4	34/10
			12/70	32/50		
Li et al. (20)	China	RC	47	40.2 (18–70)	28/2	19/7
			0/47	20/27		
Wan et al. (21)	China	RC	38	39 (12–59)	35/2	3/0
			1/37	9/29		
Zheng et al. (22)	China	RC	154 (2 aneurysm obliteration)	33.95 ± 10.47 (22–61)	124/15	28/6
			10/144	52/102		
Zhang et al. (23)	China	RC	37	50.3	29/5	8/4
			0/37	23/14		
Yang et al. (24)	China	RC	89	48 (27–66)	63/12	26/18
			0/89	50/39		
Jang et al. (25)	Korea	RC	96	NA	49/3	47/7
			0/96	NA		
Jiang et al. (26)	China	RC	40	NA	16/0	24/3
			0/40	NA		
Li et al. (27)	China	RC	52	50.7	28/0	24/5
			NA	31/23		
Liu et al. (28)	China	RC	103	38.04 ± 0.52 (32–40)	57/10	46/22
			0/103	48/55		
Present casesat our institution	China	RC	24	42 ± 11.8	12/0	12/2
			0/24	11/13		

S, surgical; NS, nonsurgical; ys, years; RV, revasculation; RB, rebleeding; CO, conservative; NA, not available; RC, retrospective cohort; MPRCT, multicentered prospective randomized, controlled trial; NOPs, number of patients.

**Table 2:** Study characteristics, ischemic event, and mortality in conservative and revascularization groups.

**References**	**Bypass approach**	**Follow-up**	**NOPs (RV/IS)**	**NOPs (CO/IS)**	**NOPs (RV/DE)**	**NOPs (CO/DE)**
Fujii et al. (10)	STA–MCA and/or EMAS or EDAS	NA	NA	NA	NA	NA
Ikezaki et al. (11)	indirect, STA–MCA direct+indirect	47.6 ms	NA	NA	NA	NA
Yoshida et al. (12)	EDAS, EMS, STA-MCA, STA-MCA+EDAS	>10 ys	NA	NA	8/1	13/4
Kawaguchi et al. (13)	STA–MCA, EDAS	8 ys (0.8-15.1)	11/1	11/4	NA	NA
Duan et al. (14)	EDAS	27.5 ms (6–64)	NA	NA	59/0	2/2
Zhao et al. (15)	STA-MCA	S 2.52, NS 1.6 ys	10/1	13/2	NA	NA
Choi et al. (16)	STA-MCA, EDAGS EDAMS, EMS	55.4 ms (12–105)	NA	NA	35/1	9/1
Liu et al. (17)	STA-MCA, EDAS STA-MCA+EDAS	7.1 ys	NA	NA	54/2	43/4
Miyamoto et al. (18)	STA–MCA direct+indirect	5 ys	42/1	38/1	NA	NA
Chen et al. (19)	STA-MCA STA-MCA+EDAS	7.8 ys (0.6–12)	48/2	34/5	48/2	34/10
Li et al. (20)	STA-MCA EMS, EDAMS	26 ms (12–44)	NA	NA	28/0	19/4
Wan et al. (21)	EDAS	51 ms (13–125)	NA	NA	35/1	3/0
Zheng et al. (22)	STA-MCA, indirect direct+indirect	36.12 ms	124/7	28/3	124/9	28/3
Zhang et al. (23)	STA–MCA, EMS EDAMS, EDAGS direct+indirect	12–97 ms	NA	NA	29/1	8/2
Yang et al. (24)	STA-MCA+EDMS EDMS	RV 19ms (12–24) CO 10 ms (8–15)	NA	NA	NA	NA
Jang et al. (25)	STA-MCA, indirect direct+indirect	6ys	49/2	47/10	49/3	47/3
Jiang et al. (26)	STA-MCA+EDMS	1 y	NA	NA	NA	NA
Li et al. (27)	STA-MCA EDAS	5 ys	NA	NA	28/0	24/5
Liu et al. (28)	EDMS, EMAS	1–6 ms	NA	NA	NA	NA
Present cases at our institution	EMS, STA-MCA, indirect bypass, dural inversion	51 ± 30 ms (14–97)	12/0	12/1	12/0	12/2

STA–MCA, superficial temporal artery–middle cerebral artery bypass surgery; EMAS, encephalo-myo-aterio-synangiosis; EDAS, encephalo-duro-aterio-synangiosis; EMS, encephalo-myo-synangiosis; EDAGS, encephalo-duro-arterio-galeo-synangiosis; EDAMS, encephalo-duro-arterio-myo-synangiosis; EDMS, encephalo-duro-myo-synangiosis; ms, months; ys, years; RV, revasculation; CO, conservative; NA, not available; NOPs, number of patients.

### 2.4. Statistical analysis

The data available from the selected studies were imported into Review Manager, version 5.3.5 (The Cochrane Collaboration), for quantitative analysis. Odds ratios (ORs) with 95% CIs were calculated in Review Manager. The heterogeneity between the studies was considered valid with a *P* < 0.05 in Cochran's Q-test. In the Higgins inconsistency index (I^2^) test, the degrees of heterogeneity were as follows: 0% to 40% might not be important; 30% to 60% may represent moderate heterogeneity; 50% to 90% may represent substantial heterogeneity; and 75% to 100% may represent considerable heterogeneity (29). Whether a random-effect or fixed-effect meta-analysis was performed depended on the heterogeneity among studies. The publication bias was tested by utilizing a funnel plot in our meta-analysis.

## 3. Results

### 3.1. Baseline characteristics

A total of 26 patients with HMMD were treated at our institution between May 2013 and May 2022, and two were lost to follow-up. Among the other 24 patients, 12 underwent revascularization, and the other 12 received conservative treatment. The mean follow-up time was 51 months (14–97), during which no rebleeding, ischemic event, or rebleeding-related mortality occurred in 12 patients who underwent revascularization, whereas in the conservative group, rebleeding occurred in two patients (16.7%), an ischemic event in one patient (8.3%), and death in two patients (16.7%) (Table 3).

**Table 3:** Baseline characteristics of our present patients.

**Characteristics**	**Total**
Age in years (mean ± SD)	42 ± 11.8
Gender (Female/Male)	13/11
Revascularization/Conservative	12/12
Revascularization procedure	2 craniectomy and EMS,10 STA-MCA, indirect bypass, and dural inversion
Rebleeding	Revascularization 0/conservative 2
Ischemic event	Revascularization 0/conservative 1
Mortality	revascularization 0/conservative 2
Follow up duration	51 ± 30 months (14–97)
MRS	0.72 ± 1.1

SD, standard deviation; mRS, modified Rankin Score.

Among the 20 studies carried out in East Asia, including our institution's consecutive case series, five studies (25%) were conducted in Japan, 13 (65%) in China, and 2 (10%) in Korea, respectively, and there were 11 (55%) studies comprising adult patients only, 6 (30%) comprising adult and pediatric patients, and 3 (15%) that did not clearly mention the study population. In total, 19 studies were retrospective cohorts, and 1 was a multicenter prospective randomized controlled trial. The follow-up duration ranged from 1 month to >10 years. Among the 20 studies reviewed, direct (STA-MCA) and indirect bypass procedures were performed in 17 (85%) studies; indirect bypass alone was used in the other three studies, which included encephalic-myo-spongiosis (EMS) (12, 16, 20, 23), encephalo-duro-aterio-synangiosis (EDAS) (10, 12–14, 17, 19, 21, 27), encephalo-duro-myo-synangiosis (EDMS) (24, 26, 28), and encephalo-myo-aterio-synangiosis (EMAS) (10, 28), encephalo-duro-arterio-galeo-synangiosis (EDAGS) (16, 23), and encephalo-duro-arterio-myo-synangiosis (EDAMS) (16, 20, 23).

### 3.2. Rebleeding

**Figure 2:**
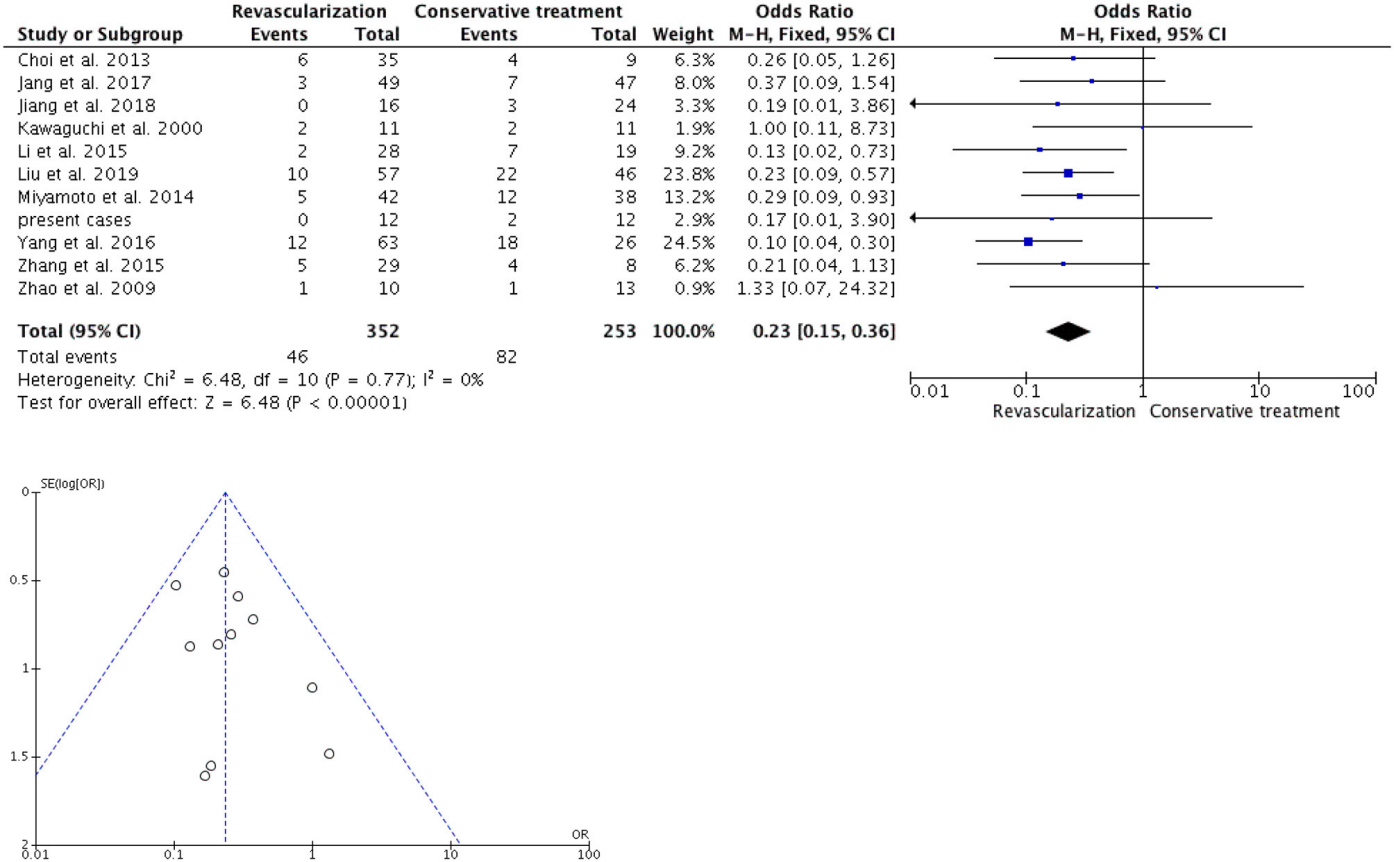
In a fixed-model, the forest plot of odd ratios for rebleeding occurred in 11 adult HMMD studies (including our present cases). Patients with revascularization had less rebleeding compared with conservative treatment (OR, 0.23; 95% CI, 0.15–0.36; *P* < 0.00001). Funnel plot for the 11 adult studies included in this meta-analysis. M-H, Mantel-Haenszel; CI, confidence interval.

**Figure 3:**
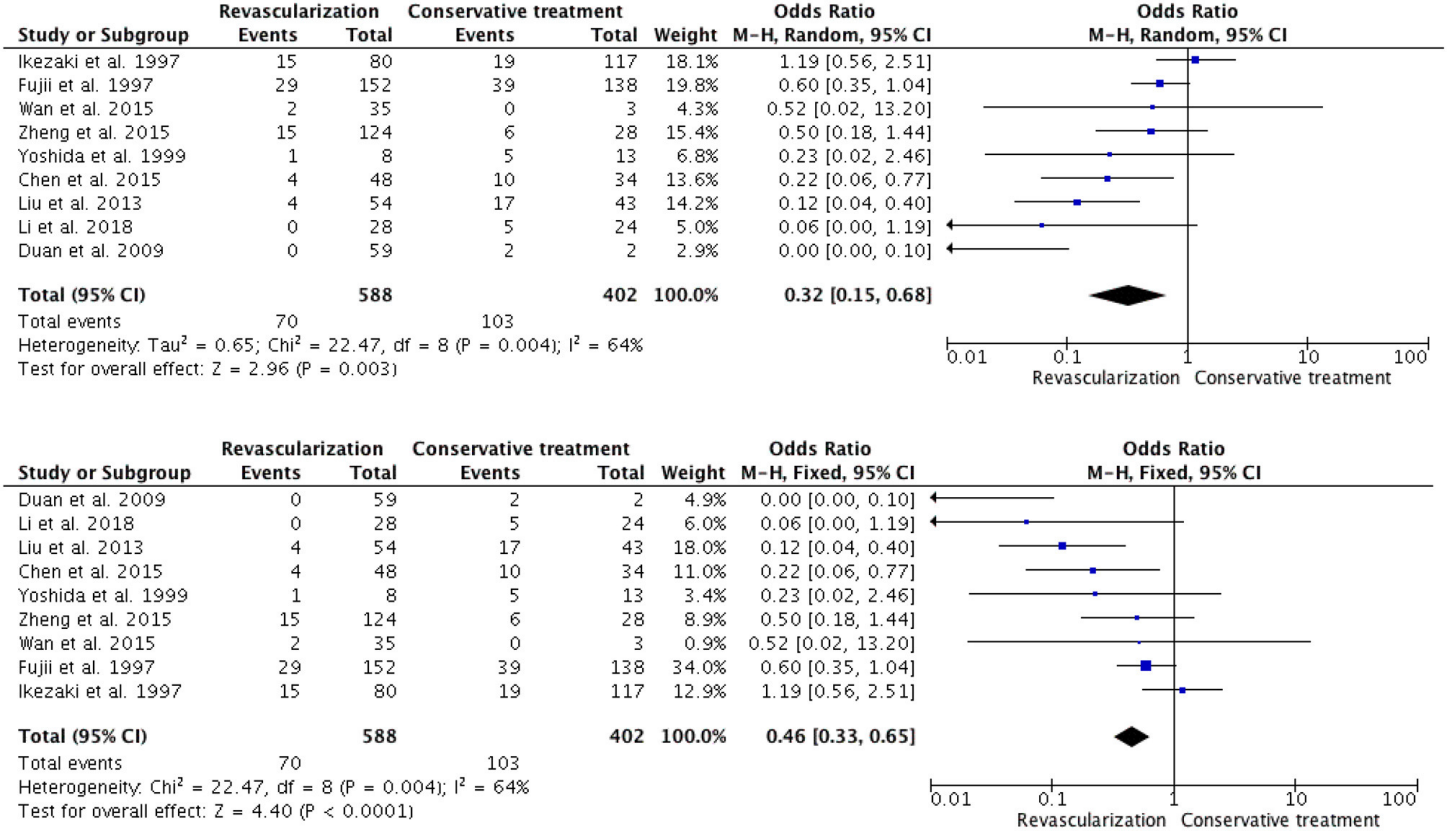
In a random and fixed-model, the forest plot of odd ratios for rebleeding occurred in nine adult/pediatric and not specifically mentioned patients HMMD studies. Patients with revascularization had less rebleeding compared with conservative treatment (OR, 0.32; 95% CI, 0.15–0.68; *P* = 0.003, and OR, 0.46; 95% CI, 0.33–0.65; *P*< 0.0001, respectively).

In the 20 studies, including our institution's series, there were a total of 940 patients who underwent revascularization, among whom 116 (12.3%) patients experienced rebleeding, whereas 185 (28.2%) of the 655 patients who received conservative treatment experienced rebleeding. The rebleeding rate in the 11 adult revascularization groups was 13.1% (46 out of 352 patients), whereas, in the conservative treatment group, 82 out of 253 patients (32.4%) experienced rebleeding. The heterogeneity testing revealed no heterogeneity among these studies (I^2^ = 0%, *P* = 0.77). The meta-analysis showed a pooled OR of 0.23 (95% CI 0.15-0.36; *p* < 0.00001) (Figure 2) in the Mantel-Haenszel fixed-effects model. Of the nine adult/pediatric and not specifically mentioned patients, 70 (11.9%) out of the 588 patients experienced rebleeding, and 103 (25.6%) out of 402 patients were in the conservative treatment group. The patients who underwent revascularization experienced significantly less rebleeding than those who received conservative treatment (OR, 0.32; 95% CI, 0.15–0.68; *P* = 0.003, and OR, 0.46; 95% CI, 0.33–0.65; *P* < 0.0001, in a random and fixed-effects model, respectively) (Figure 3). Compared with conservative treatment, surgical revascularization significantly reduced the incidence of rebleeding in HMMD patients.

### 3.3. Ischemic events

**Figure 4:**
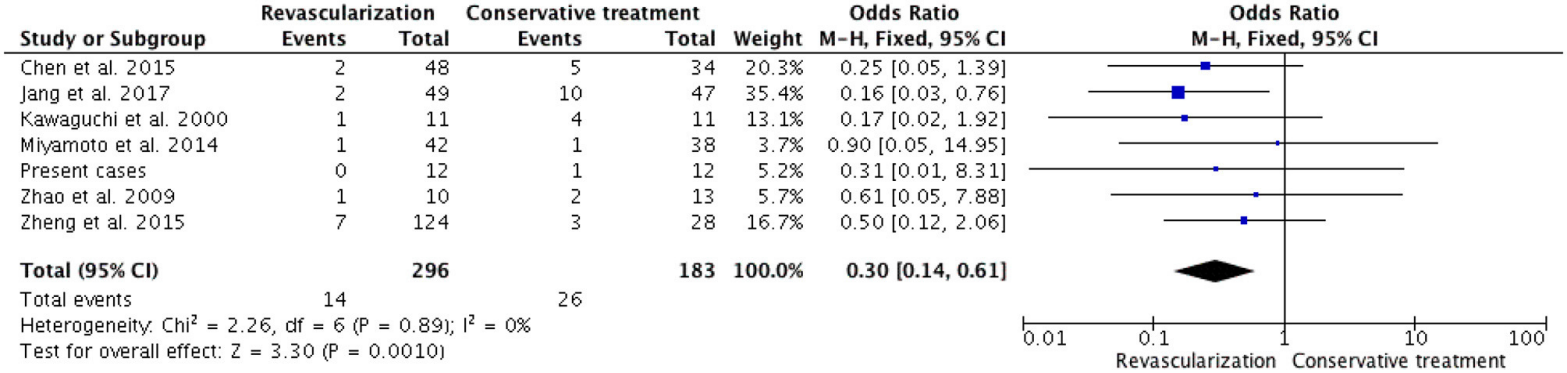
In a fixed-model, the forest plot of ORs for ischemic events in 7 adult/pediatric HMMD studies (including our present cases). Patients with revascularization had less ischemic events compared with conservative treatment (OR, 0.30; 95% CI, 0.14–0.61; *P* = 0.001).

**Figure 5:**
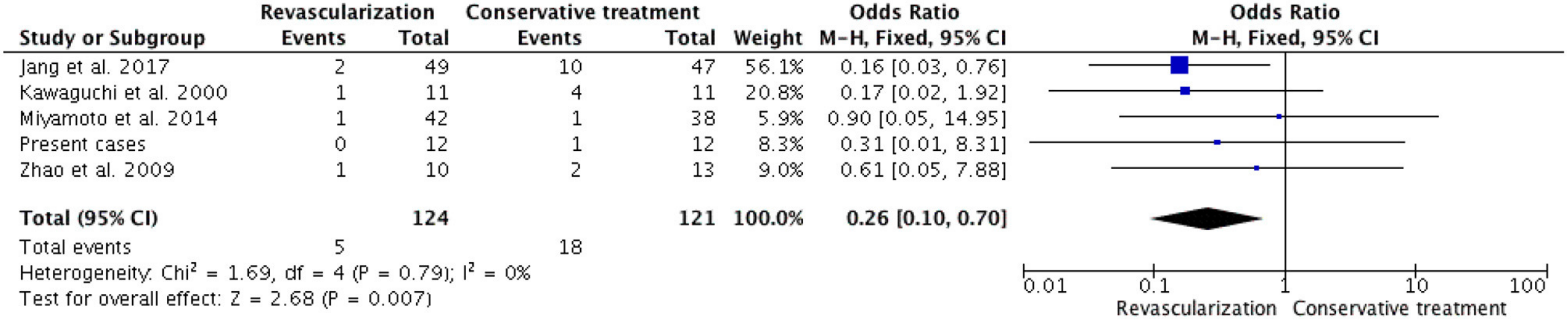
In a fixed-model, the forest plot of ORs for ischemic event occurred in five adult HMMD studies (including our present cases). Patients with revascularization had less ischemic events compared with those with conservative treatment (OR, 0.26; 95% CI, 0.10–0.70; *P* = 0.007).

Among 20 studies, there were seven studies (including our present cases) related to the post-surgical ischemic event. There were 14 cases (4.7%) of complicated postoperative ischemic events in patients who underwent revascularization and 26 cases (14.2%) were observed among 183 patients who received conservative treatment. Patients who underwent revascularization experienced fewer ischemic events compared with those who received conservative treatment (OR, 0.30; 95% CI, 0.14–0.61; *P* = 0.001, Figure 4). Among the seven studies, five studies comprised adult patients only; a total of 124 patients underwent revascularization, among whom 5 (4.0%) experienced ischemic events, whereas 18 (14.9%) of the 121 patients who received conservative treatment experienced ischemic events. There was no heterogeneity between the results of the five studies (I = 0%, *P* = 0.79), and the fixed-effects model was selected for meta-analysis. A comparison of the revascularization group with the conservative treatment group in a fixed-effects meta-analysis showed a pooled OR of 0.26 (95% CI 0.10-0.70; *p* = 0.007, Figure 5). As mentioned above, in adult and pediatric HMMD patients, the revascularization procedure provided a significant advantage over conservative treatment in reducing the incidence of ischemic events.

### 3.4. Mortality

**Figure 6:**
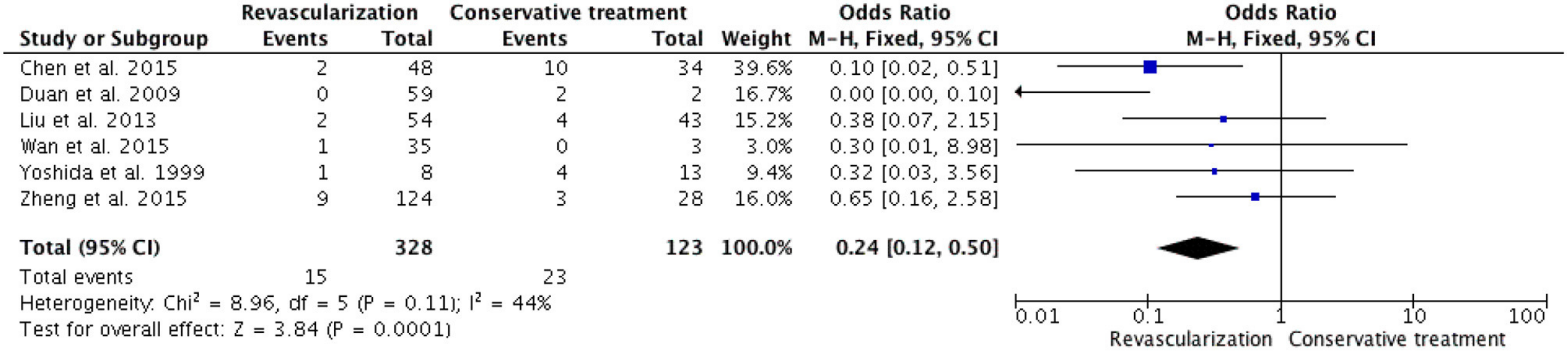
In a fixed-model, the forest plot of ORs for mortality occurred in six adult/pediatric patients HMMD studies. Patients with revascularization had less mortality compared with those with conservative treatment (OR, 0.24; 95% CI, 0.12–0.50; *P* = 0.0001).

**Figure 7:**
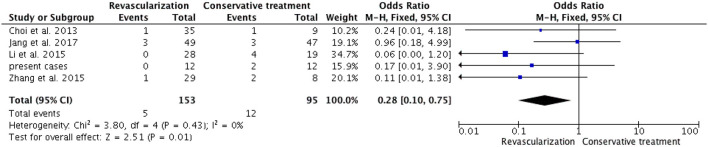
In a fixed-model, the forest plot of ORs for mortality occurred in five adult HMMD studies (including our present cases). Patients with revascularization had less mortality compared with those with conservative treatment (OR, 0.28; 95% CI, 0.10–0.75; *P* = 0.01).

Among the six studies that included both adult and pediatric patients, there were 15 deaths (4.6%) due to rebleeding among 328 patients who underwent revascularization, whereas the mortality among patients who received conservative treatment was 18.7% (23/123). The meta-analysis showed a pooled OR of 0.24 (95% CI 0.12-0.50; *P* = 0.0001) (Figure 6) in the Mantel-Haenszel fixed-effects model, which revealed a significant reduction in mortality associated with revascularization surgery compared to conservative treatment in mixed adult/pediatric patients. Among the five studies that included only adult patients, the mortality rate was significantly lower in the revascularization group [3.3% (5/153)] than in the conservative treatment group [12.6% (12/95)]. The meta-analysis showed a pooled OR of 0.28 (95% CI 0.10-0.75; *P* = 0.01) (Figure 7) in the Mantel-Haenszel fixed-effects model. The results presented above indicate that revascularization is associated with lower mortality rates than conservative treatment.

## 4. Discussion

The HMMD patients experienced a significantly higher frequency of intracranial hemorrhage than ischemic events, with this difference becoming more pronounced over longer periods of follow-up (30). In Yamada et al.'s (31) study, there was no statistically significant difference in the hemorrhagic recurrence rate between the patients who underwent revascularization and those who received conservative treatment. Lee SB et al. (32) revealed that direct and combined revascularization statistically prevented ischemic stroke recurrence in adult ischemic MMD patients, whereas no statistically significant difference was found in reducing the incidence of re-hemorrhage in the HMMD adult patients who underwent revascularization surgery. To the best of our knowledge, there is still no meta-analysis study on HMMD rebleeding, ischemic events, or mortality differences between the conservative and revascularization groups in the East Asian population. The debate regarding a superior treatment option between revascularization and conservative treatment in HMMD is ongoing. An ideal treatment approach for any medical condition should prioritize strategies that result in less rebleeding, fewer ischemic events, and fewer mortalities.

### 4.1. Rebleeding

Initial and recurrent bleeding episodes in patients with moyamoya disease occur mainly in adult patients, resulting in neurological deficits and reduced quality of life. Hemorrhage is typically caused by the rupturing of fragile perforator vessels, proliferative collateral vessels, and concomitant micro-aneurysms, which are all believed to be induced by elevated autoantibodies or/and hemodynamic stress that leads to apoptosis (33, 34). In Takahashi et al.'s report on HMMD, an independent rebleeding risk factor was a hemodynamic failure, and a significant preventive effect was obtained by the direct bypass procedure in the hemodynamically disrupted hemispheres (35). Yamada S et al. found that the estimated rebleeding rate of HMMD was 9.4 ± 3.0%/3 years and 10.9 ± 3.3%/5 years, respectively (31). In the 11 adult groups of our meta-analysis, 13.1% of cases (46/352) who underwent the revascularization procedure experienced rebleeding, whereas 32.4% of cases (82/253) in conservative treatment experienced hemorrhage. In the present 20 adult/pediatric patients' groups, 12.3% (116/940) and 28.2% (185/655) rebleeding occurred in the surgical and no surgical groups, respectively, with follow-up durations ranging from 1 month to >10 years. In Kim et al.'s adult study, the estimated rebleeding rate was 16.9%/person at five years and 26.3%/person at 10 years (36), which was similar to the rate of our adult/pediatric patients' groups with conservative treatment. The surgical revascularization in MMD is deemed to reduce persistent hemodynamic stress on fragile collateral vessels or/and accompanying aneurysms, resulting in a significant regression of these fragile vessels. The resumed blood flow and vascular reserve capability improve hemodynamic stabilization. However, there is still no ideal revascularization modality for HMMD, and there is also no optional medicine that can stop or reverse the insidious and progressive disease course. Different kinds of implanted tissues used in indirect bypass surgery were reported: encephalo-myo-synangiosis (EMS), encephalo-myo-arterio-synangiosis (EMAS), encephalo-duro-arterio-synangiosis (EDAS), encephalo-duro-myo-synangiosis (EDMS), encephalo-duro-myo-arterio-synangiosis (EDAMS), and encephalo-duro-arterio-galeo-synangiosis (EDAGS) were performed in studies included in the present review, and the previous studies showed that about 50–80% adult patients improved after indirect bypass procedure (37, 38). Among the reviewed 20 studies, the STA-MCA bypass procedure was performed in 17 studies (85%), and in the 11 studies with adult patients only, the direct bypass surgery was performed in 10 studies (90.9%). The direct bypass results in immediate cerebral hemodynamic improvement, and the direct bypass comprises the main treatment option for the reviewed studies, especially in adult patients. At the same time, an indirect bypass was also used as an important supplementary treatment in all 11 adult studies, of which an indirect bypass was chosen as the only treatment option in one study. The indirect bypass was accompanied by direct bypass surgery. This may be because the chronically induced angiogenesis resulting from the indirect bypass procedure will continue to contribute to further hemodynamic improvement after the immediate blood flow augmentation by direct bypass surgery. The indirect bypass is encouraging, with collateral arterial neoangiogenesis, age-dependent cerebrovascular plasticity, and low perioperative risk. Direct bypass is always challenging in pediatric or adult patients with advanced-stage MMD due to the lower bypass patency rates and caliber mismatch between donor and recipient vessels. The direct and indirect bypass procedures are reciprocal and synergistic in improving cerebral hemodynamics.

### 4.2. Ischemic event

Among the 20 reviewed studies, seven involved mixed adult/pediatric patients with post-surgical ischemic events, among which 14 cases (4.7%) were found to be complicated by postoperative ischemic events in 296 patients who underwent revascularization and 26 cases in 183 patients (14.2%) who received conservative treatment. Patients who underwent revascularization were significantly less likely to result in ischemic events than those with conservative treatment (OR, 0.30; 95% CI, 0.14–0.61; *P* = 0.001). Among the five adult patient-only studies, there were 5 in 124 (4.0%) revascularization patients with ischemic events, 18 in 121 (14.9%) conservatively treated patients, and adult patients who had undergone revascularization had fewer ischemic events compared with those with conservative treatment (OR, 0.26; 95% CI, 0.10–0.70; *P* = 0.007). In the study of Kim et al., 5.7% of patients (4/70, 2 with combined surgery, and 2 with indirect) experienced postoperative infarction, and the other four ischemic strokes occurred in the conservative treatment group, whose postoperative infarction rate was similar to our review (36). Kim et al. (36) also found that the ischemic events in HMMD patients were minor strokes, whereas, in our review, there were two adult patients with complete ischemic stroke and right hemiplegia, respectively (15, 18). The progressive cerebral arterieal occlusive disease and poorly developed collateral vessels always contribute to a postoperative ischemic event (39). The revascularization procedure has been shown to increase cerebral blood flow and improve cerebral vascular reserve, leading to enhanced cerebral hemodynamics and a reduction in cerebral ischemic events. On the contrary, conservative treatment with antiplatelet agents showed no potential benefit in preventing further strokes because of the mismatch between the pathophysiological changes of MMD and the pharmacological mechanism of aspirin.

Of the 20 studies included in our meta-analysis, direct bypasses (STA-MCA) were performed in 17 studies (85%), and indirect bypass was performed in only three studies (15%) (5, 12, 19). Moreover, direct bypass was the more preferable choice in adult patients due to its immediate increase in blood flow to the cerebral hemodynamic deficit area. In the acute stage after indirect bypass, there is a dangerous time window during which swelling of the temporal muscle, brain protrusion from the craniotomy site, and disruption of previous collateral circulation all potentially reduce cerebral blood flow, especially in adult patients, which can result in postoperative ischemic events (40).

### 4.3. Mortality

The cause of death in HMMD patients is mostly due to intracranial hemorrhage, and the previously reported mortality rate ranged from 6.8 to 28.6% (41–43). In our review, the mortality rate in six mixed adult/pediatric patient studies with revascularization (4.6%, 15/328) was significantly lower than those who received conservative treatment (18.7%, 23/123) (OR, 0.24; 95% CI, 0.12–0.50; *P* = 0.0001), and in the five studies with adult patients only, similar results were obtained (3.3% (5/153) versus 12.6% (12/95), OR, 0.28; 95% CI, 0.10–0.75; *P* = 0.01). The lower mortality rate in the adult studies, as compared with that of the mixed adult/pediatric studies, indicates that the mortality rate may be lower in adults than in pediatric patients. Sang-Hyuk et al. reported that adult HMMD patients had the worst survival outcomes, and the crude mortality for 10 years was 34.7% in hemorrhagic adult South Korean MMA patients (44), which is more than twice the mortality rate of our review. The patients with recurrent hemorrhage had an 11.04-fold risk of death compared to those without it, and the main cause of death in HMMD patients was rebleeding (45). As found in our review, the revascularization procedure significantly prevented rebleeding in HMMD patients, and the mortality rate associated with rebleeding decreased accordingly.

## 5. Limitations

First, different neurosurgical centers with different patient volumes have varying levels of experience, and the studies included in the review ranged over a long period of time, within which improvements were achieved in the diagnosis and treatment of MMD. Second, there are many kinds of revascularization procedures and different combinations of them in the reviewed studies, such as STA–MCA, EMS, EDAS, EDMS, EMAS, EDAGS, and EDAMS; however, the effect of each revascularization modality alone on the HMMD outcomes has not yet been fully explored or understood. Finally, despite the relatively small sample size of pediatric patients in our review, different cerebral hemodynamic responses to the revascularization procedure between adults and pediatric patients should not be ignored.

## 6. Conclusion

Direct revascularization, indirect bypass, and a combination of these approaches represent the mainstay treatment of HMMD, and an HMMD prognosis can be improved by surgical revascularization in terms of rebleeding, ischemic events, and mortality in East Asian Countries. Future studies may be necessary to confirm these findings, and the impact of each type of revascularization modality alone on HMMD requires future investigation and clarification.

## Data availability statement

The original contributions presented in the study are included in the article/supplementary material, further inquiries can be directed to the corresponding author.

## Author contributions

X-HZ: conceptualization, manuscript review, and editing. J-HH: writing the initial draft. X-SZ and JZ: application of statistical to analyze study data. C-jW: data collection. Y-PD and WT: visualization/data presentation. All authors contributed to the article and approved the submitted version.
